# Identification of Differentially Expressed Genes during *Bacillus subtilis* Spore Outgrowth in High-Salinity Environments Using RNA Sequencing

**DOI:** 10.3389/fmicb.2016.01564

**Published:** 2016-10-06

**Authors:** Katja Nagler, Antonina O. Krawczyk, Anne De Jong, Kazimierz Madela, Tamara Hoffmann, Michael Laue, Oscar P. Kuipers, Erhard Bremer, Ralf Moeller

**Affiliations:** ^1^Space Microbiology Research Group, Radiation Biology Department, Institute of Aerospace Medicine, German Aerospace CenterCologne, Germany; ^2^Department of Molecular Genetics, Groningen Biomolecular Sciences and Biotechnology Institute, University of GroningenGroningen, Netherlands; ^3^Advanced Light and Electron Microscopy, Center for Biological Threats and Special Pathogens, Robert Koch InstituteBerlin, Germany; ^4^Laboratory of Microbiology, Department of Biology, Philipps-University MarburgMarburg, Germany

**Keywords:** *B. subtilis* spore germination, outgrowth, ripening, high salinity, osmotic stress, NaCl, RNA-seq

## Abstract

In its natural habitat, the soil bacterium *Bacillus subtilis* often has to cope with fluctuating osmolality and nutrient availability. Upon nutrient depletion it can form dormant spores, which can revive to form vegetative cells when nutrients become available again. While the effects of salt stress on spore germination have been analyzed previously, detailed knowledge on the salt stress response during the subsequent outgrowth phase is lacking. In this study, we investigated the changes in gene expression during *B. subtilis* outgrowth in the presence of 1.2 M NaCl using RNA sequencing. In total, 402 different genes were upregulated and 632 genes were downregulated during 90 min of outgrowth in the presence of salt. The salt stress response of outgrowing spores largely resembled the osmospecific response of vegetative cells exposed to sustained high salinity and included strong upregulation of genes involved in osmoprotectant uptake and compatible solute synthesis. The σ^B^-dependent general stress response typically triggered by salt shocks was not induced, whereas the σ^W^ regulon appears to play an important role for osmoadaptation of outgrowing spores. Furthermore, high salinity induced many changes in the membrane protein and transporter transcriptome. Overall, salt stress seemed to slow down the complex molecular reorganization processes (“ripening”) of outgrowing spores by exerting detrimental effects on vegetative functions such as amino acid metabolism.

## Introduction

In its natural habitat, the soil bacterium *Bacillus subtilis* is frequently confronted with fluctuating environmental conditions and has therefore evolved a broad range of elaborate stress responses (Marles-Wright and Lewis, 2007, 2010; Lopez et al., 2009; Schultz et al., 2009). Two common environmental stresses in soil are changes in osmolality and limitation of nutrient availability (Wood et al., 2001; Bremer, 2002; Nicholson, 2002).

When soil desiccation creates hyperosmotic conditions, cells have to adjust their internal osmolality to avoid water efflux and plasmolysis (Wood et al., 2001; Hoffmann and Bremer, 2016). In a first response, *B. subtilis* cells quickly take up large amounts of K^+^ via the KtrAB and KtrCD transport systems to restore internal osmotic pressure (Whatmore et al., 1990; Holtmann et al., 2003). However, prolonged high intracellular K^+^ concentrations are not compatible with various cellular functions (Whatmore et al., 1990; Record et al., 1998). Therefore, *B. subtilis* subsequently replaces K^+^ by compatible solutes, highly soluble organic compounds that do not disturb cell physiology, to adjust its intracellular osmotic potential (Whatmore et al., 1990; Kempf and Bremer, 1998). Compatible solutes can either be synthesized (*de novo* or from precursors) or taken up from the environment via five osmotically inducible osmoprotectant uptake transporters (OpuA, OpuB, OpuC, OpuD, OpuE) that differ in their affinities and substrate specificities (Kempf and Bremer, 1998; Hoffmann and Bremer, 2016). The most important compatible solutes for *B. subtilis* are glycine betaine (GB) and proline (Hoffmann and Bremer, 2016).

Depending on how salt stress is imposed, *B. subtilis* cells can react in distinct manners (Spiegelhalter and Bremer, 1998; Steil et al., 2003; Young et al., 2013). When *B. subtilis* is subjected to a sudden osmotic up-shock, the σ^B^-governed general stress response is activated (Spiegelhalter and Bremer, 1998; Nannapaneni et al., 2012; Young et al., 2013). In contrast, upon incremental and sustained salt stress, cells activate a specific osmotic stress response under the regulation of the house-keeping sigma factor σ^A^ (Spiegelhalter and Bremer, 1998; Steil et al., 2003; Young et al., 2013; Hoffmann and Bremer, 2016). Nevertheless, it is still not understood how increases in the environmental osmolality are perceived and how this information is processed to adjust gene expression according to the cells' needs (Hoffmann and Bremer, 2016).

A different strategy of *B. subtilis* to cope with environmental (albeit not osmotic) stress is sporulation: upon nutrient depletion *B. subtilis* can form dormant spores that are highly resistant against a broad range of environmental extremes such as heat, desiccation, and chemicals (Ruzal et al., 1998; Nicholson et al., 2000; Setlow, 2006, 2013). A dormant spore consists of a dehydrated spore core (analogous to a growing cell's protoplast) that is enveloped by a dense inner membrane, a germ cell wall, a cortex, and a proteinaceous spore coat (Setlow, 2006). Although spores can remain dormant for extended periods of time, they can convert back to vegetative cells via a process called germination when nutrients become available (Nicholson, 2002; Setlow, 2013). Throughout germination, spores release ions and Ca^2+^-dipicolinate (Ca^2+^-DPA), hydrolyze their cortex, and rehydrate, which causes the loss of their refractivity and resistance properties (reviewed in Setlow, 2013). After germination is completed, the former spores enter a phase called outgrowth, which is defined as the time period between the onset of metabolic activity and the first cell division (Setlow, 2003; Keijser et al., 2007). Throughout outgrowth the germinated spores undergo molecular reorganization (“ripening”), escape from their spore coats, and elongate (Keijser et al., 2007; Segev et al., 2013; Setlow, 2013; Sinai et al., 2015). Important events in early outgrowth are the generation of ATP, nucleotides, and amino acids from endogenous resources, as well as the onset of macromolecular synthesis (Paidhungat and Setlow, 2002; Setlow, 2003; Keijser et al., 2007; Sinai et al., 2015). On the genomic level, the importance of σ^A^ as well as the temporal activation of at least 30% of all *B. subtilis* genes during a well-regulated spore outgrowth program have been reported (Horsburgh et al., 2001; Keijser et al., 2007). Correspondingly, outgrowing spores synthesize more than 650 different proteins before entering vegetative growth (Sinai et al., 2015).

While the effects of high salinity on *B. subtilis* spore germination have been analyzed previously (Nagler et al., 2014, 2015; Nagler and Moeller, 2015), detailed knowledge on the salt stress response during the subsequent outgrowth phase, especially on a transcriptomic level, is lacking. Therefore, we investigated changes in the gene expression profile of outgrowing *B. subtilis* spores in the presence of 1.2 M NaCl by RNA sequencing (RNA-seq). A key result of our study was the observation that the transcriptional profile of salt-stressed outgrowing spores exhibits many similarities to continuously salt-stressed vegetative cells, whereas the σ^B^-controlled general stress regulon was not engaged.

## Materials and methods

### Spore production and purification

Spores of *B. subtilis* 168 (*trpC2*; DSM402) were produced in liquid cultures of modified Schaeffer's Sporulation Medium with glucose (2x SG; as described in Nicholson and Setlow, 1990). All chemicals were ordered from Sigma-Aldrich (St. Louis, MO, USA). The sporulation cultures were incubated at 37°C for 48 h in a shaking incubator (200 rpm). Spores were harvested, washed with distilled, sterile water at least seven times, and retrieved by centrifugation. The purity of the spore stocks, as checked by phase-contrast microscopy, was ≥99%. Spores were stored in distilled water in screw-capped glass tubes at 4°C until use.

### Spore germination and outgrowth experiments

Spores were heat activated at 70°C for 30 min in order to ensure synchronized germination. Germination and outgrowth experiments were performed in germination media composed of Spizizen Minimal Medium (SMM; as described in Nicholson and Setlow, 1990) with or without 1.2 M NaCl, which additionally contained 50.5 mM D-glucose, 0.5 mM L-tryptophan, and 10 mM of the germination trigger L-alanine.

The transcriptomics outgrowth experiments were performed in 45 ml germination medium (500 ml flasks). The medium was inoculated with 1.2 × 10^10^ heat-activated spores (in total) and 15 ml samples were withdrawn at 30, 60, and 90 min after inoculation. The samples were immediately mixed with ice-cold killing buffer (Nicolas et al., 2012) and washed with ice cold water by centrifugation (1 min at 10,000 x *g* at 4°C). The pellet was resuspended in 400 μl ice-cold LETS buffer (0.1 M LiCl, 0.01 M Na_2_EDTA, 0.1 M Tris-HCl pH 7.4, 0.2% SDS), transferred to a pre-cooled Lysing Matrix B tube (MP Biomedicals, Santa Ana, CA, USA) containing 500 μl phenol:chloroform (1:1) and 25 μl 10% SDS, and used for RNA isolation. For the dormant spore RNA samples, spore suspensions were also heat-treated for consistency. Subsequently they were centrifuged, the pellets were resuspended in LETS buffer, and transferred to Lysing Matrix B tube for RNA isolation. The transcriptomics outgrowth experiments were performed in duplicate using two independent spore batches.

For spectrophotometric measurements, germination was carried out in triplicate in 96-well plates, each containing 200 μl of germination media. Each well was inoculated with 40 μl heat-activated spores to a starting optical density of ca. 0.5 at 600 nm (OD_600nm_) corresponding to a total of ca. 4 × 10^7^ spores per well. The plate was incubated at 37°C in a multi-plate reader (ELx808IU, BioTek, Bad Friedrichshall, Germany) that read the OD_600nm_ of the culture, with 5 s of shaking before all readings. The OD_600nm_ data was normalized by division of each reading by the first measured value (*t*_0min_), yielding the relative OD_600nm_ given in %. A 60% decrease in relative OD_600nm_ corresponds to germination of the whole spore population (Atluri et al., 2006; Nagler et al., 2014).

### Microscopy

For scanning electron microscopy (SEM) of outgrowing spores, dormant spores were germinated as described above. Samples were withdrawn 30, 60, and 90 min after germination initiation, washed with distilled water and fixed in 2.5% glutaraldehyde. Fixed samples were washed with distilled water, adsorbed to an Alcian blue-coated cover slip, and stored in 2.5% glutaraldehyde (in 0.05 M HEPES) overnight. Then, samples were washed with distilled water, treated with 1% osmium, washed again, dehydrated with increasing concentrations of ethanol, and dried by critical-point drying (Emitech K850, UK). Dried samples were sputter-coated with 3 nm Au/Pd (Polaron E5100) and analyzed by SEM (Gemini 1530, Carl Zeiss Microscopy GmbH, Germany) using an acceleration voltage of 5 kV and the in-lens secondary electron detector.

**Figure 1:**
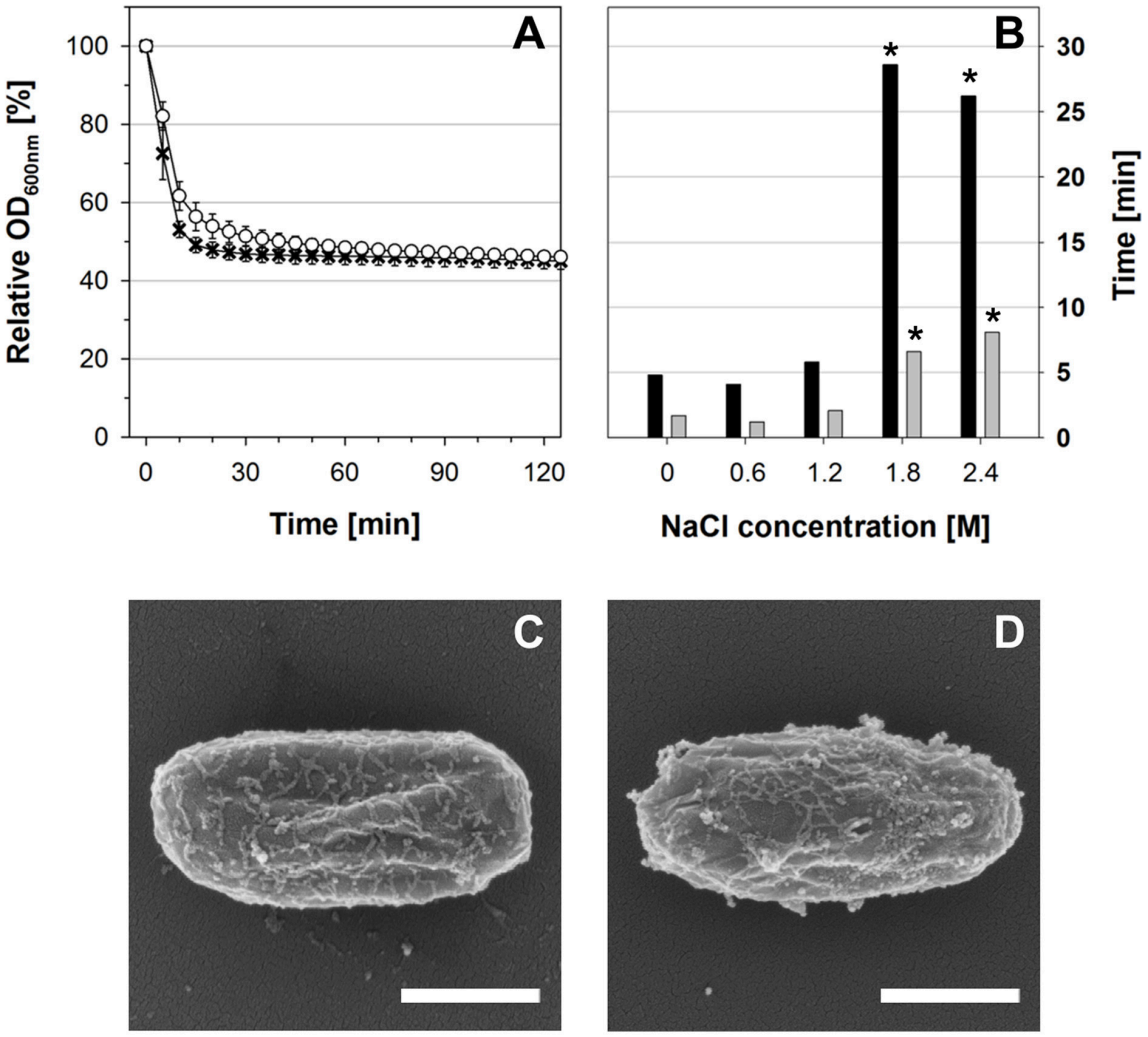
**(A)** Spore germination and outgrowth profiles in SMM supplemented with glucose, L-tryptophan, and L-alanine as measured by OD_600nm_. The medium contained either 1.2 M NaCl (white circles) or no NaCl (black crosses). **(B)** Single-spore live cell imaging analyses. The times required for start of refractivity loss (black bars) and duration of refractivity loss (gray bars) at different NaCl concentrations are shown as median-values (*n* ≥ 74 spores). Asterisks indicate significant (*p* ≤ 0.01) differences to the values at 0 M NaCl. **(C,D)** SEM pictures 90 min after germination initiation **(C)** in the absence of NaCl or **(D)** in the presence of 1.2 M NaCl. Scale bars = 500 nm.

For live cell imaging of individual spores, dormant spores were dried in a plastic dish (μ-dish, ibidi, Germany). The dried spores were covered with germination medium (as described above) that was solidified with 1.5% agarose. Germination and outgrowth were observed by phase-contrast with a Nikon TE2000-E Eclipse microscope and a Plan Fluor 100/1.3 Oil objective. Photos were taken every 5 s and merged into time-lapse videos. Germination parameters (starting time of change from bright to dark and duration of change) were determined using ImageJ (Rasband, 1997) and are given as median-values (with spore counts ranging from 74 to 124 spores per condition) in Figure 1B.

To monitor germination by phase-contrast microscopy, spores were germinated in 96-well plates as described above. At appropriate time points, 5 μl samples were withdrawn and fixed by applying to a microscope slide coated with 1% agar. Micrographs were taken using a Zeiss fluorescence microscope (Axio Imager M2, Carl Zeiss MicroImaging GmbH, Germany) equipped with an AxioCam MRm.

### RNA isolation

RNA isolation was performed with a phenol-chloroform extraction method as follows. The samples (in Lysing Matrix B tubes, see above) were immediately disrupted using a FastPrep device (Eubio, Austria), with four subsequent disruptions (45 s at 6.5 m/s) separated by 1–2 min incubation on ice to avoid overheating. After disruption, samples were centrifuged at 4°C and the supernatant was mixed with chloroform. After centrifugation at 4°C, the RNA was precipitated by 0.3 M sodium acetate (pH 5.3) in isopropanol for 3 h on ice. The pellet was washed with 70% ethanol, dried, resuspended in nuclease-free water, and treated with a RNase-free DNase Set (Qiagen, Hilden, Germany) according to the manufacturer's manual (incubation for 1 h at 37°C). The treated samples were diluted with nuclease-free water and mixed with the same amount of phenol:chloroform:isoamylalcohol (25:24:1). After centrifugation at 4°C, the RNA was precipitated by 0.3 M sodium acetate (pH 5.3) in isopropanol overnight at 4°C. The pellet was washed with 70% ethanol, dried, and resuspended in 50 μl nuclease-free water.

### RNA sequencing and data analyses

RNA concentration of the samples was quantified using a NanoDrop 2000c instrument (Wilmington, DE, USA). Sample quality was determined with an Agilent 2100 Bioanalyzer and an Agilent RNA 6000 Nano Kit (Agilent Technologies, Waldbronn, Germany) according to the manufacturer's manual. The samples were stored at −80°C until analysis. RNA-seq was performed by the PrimBio Research Institute (Exton, PA, USA). The obtained raw data containing 4391 genes were then subjected to analyses using the webserver-based RNA-seq analysis pipeline T-Rex as described by De Jong et al. (2015). Unless noted otherwise, the transcriptomics data are expressed as the contrast of the RNA that was present in outgrowing spores in the presence of NaCl (“target”) against RNA in the absence of NaCl (“control”). T-REx includes two different significance thresholds termed “TopHits” [log_2_ fold change (log_2_FC) ≥ 2 and *p* ≤ 0.05] and “HighFold” (log_2_FC ≥ 5 and a *p* ≤ 0.01); unless noted otherwise TopHits values are shown. Additionally, the transcriptomics data was analyzed in JBrowse 1.11.6 (Skinner et al., 2009). Hierarchical clustering of transcription profiles was performed with the TIGR Multiexperiment Viewer (MeV, http://mev.tm4.org/). Functional categorization was performed according to the *Subti*Wiki platform (http://www.subtiwiki.uni-goettingen.de; Mäder et al., 2012; Michna et al., 2016). The RNA-seq data was deposited in the Gene Expression Omnibus (GEO) database (http://www.ncbi.nlm.nih.gov/geo/) under the accession number GSE81238.

## Results and discussion

### Spore germination and outgrowth at high salinity

*B. subtilis* spore germination at high salinity has previously been investigated (Nagler et al., 2014, 2015; Nagler and Moeller, 2015), but only little is known about the effects of salt stress on the transcriptional profile of outgrowing spores. In our study, spores of *B. subtilis* 168 were germinated with L-alanine in minimal medium containing either no NaCl or 1.2 M NaCl. This salt concentration was chosen in accordance with former studies on the salt stress response in vegetative *B. subtilis* cells and studies on *B. subtilis* spore germination at high salinity (Boch et al., 1994; Steil et al., 2003; Nagler et al., 2014, 2015, 2016).

In agreement with previous results (Nagler et al., 2014), the OD_600nm_ decrease of the germination culture that corresponds to germination was slightly slower in the presence of 1.2 M NaCl, but ultimately almost the complete spore population germinated successfully within 30 min (Figure 1A). Due to the low nutrient-content in the minimal medium, no growth could be observed by OD_600nm_ and phase-contrast microscopy within 2 h (Figure 1A and data not shown). Single-spore live cell imaging and student's *t*-test analysis showed that neither the starting time of the change from highly refractive to dark nor the duration of this refractivity change were significantly different in the presence or absence of 1.2 M NaCl (Figure 1B). In contrast, both processes were significantly prolonged at NaCl concentrations ≥1.8 M. Furthermore, phase-contrast microscopy and SEM revealed that stressed and non-stressed outgrowing spores had essentially the same morphology at all sample time points (30, 60, 90 min) of the transcriptomics experiment and were still encased in their spore coats after 90 min, thus resembling dormant spores by SEM (Figures 1C,D; Figure S1, and data not shown). This indicates that the spores germinated under both conditions were in their ripening phase of outgrowth, throughout which the outgrowing spores undergo molecular reorganization, but do not exhibit morphological changes (Segev et al., 2013). Altogether these data show that the transcriptomes of outgrowing spores in the presence and absence of 1.2 M NaCl can be compared to each other and reflect the impact of NaCl on gene expression within the ripening phase of outgrowth.

### Dormant spore RNA

Dormant spores contain RNA, including sporulation- and spore-related transcripts that are remnants from the spore formation process (e.g., Keijser et al., 2007; Segev et al., 2012; Bassi et al., 2016). This RNA can be degraded during early outgrowth, thus serving as a ribonucleotide reservoir for *de novo* RNA synthesis (Setlow and Kornberg, 1970; Keijser et al., 2007; Segev et al., 2012). Other transcripts may have a functional relevance, potentially being rapidly translated at the beginning of spore revival (Keijser et al., 2007; Segev et al., 2013; Sinai et al., 2015). Preliminary microarray data have suggested that degradation of spore-related RNA may be slower during outgrowth under salt stress, thereby falsely indicating these genes as “upregulated” under stress conditions (Nagler, 2012). Hence, to allow for discrimination of RNA that is newly transcribed during outgrowth from the dormant spore transcripts and to investigate the composition of the dormant spore transcriptome, we analyzed the RNA content of dormant spores.

Overall, 955 common transcripts were detected in dormant spore samples of two independent spore batches, albeit partially with low abundance (GSE81238). Yet, a high consistency of transcripts with high abundance among the two samples and a notable overlap with previous studies (Keijser et al., 2007; Segev et al., 2013) suggest that the RNA content of dormant spores was not random. In total, we found 21 out of the 25 mostly sporulation- or spore-specific transcripts that were detected by Keijser et al. (2007), as well as 103 out of 369 different dormant spore transcripts reported by Segev et al. (2012). The latter overlap of ca. 35% can still be considered substantial, regarding the different genetic background (PY79 vs. 168) and various differences in the experimental setup.

**Figure 2:**
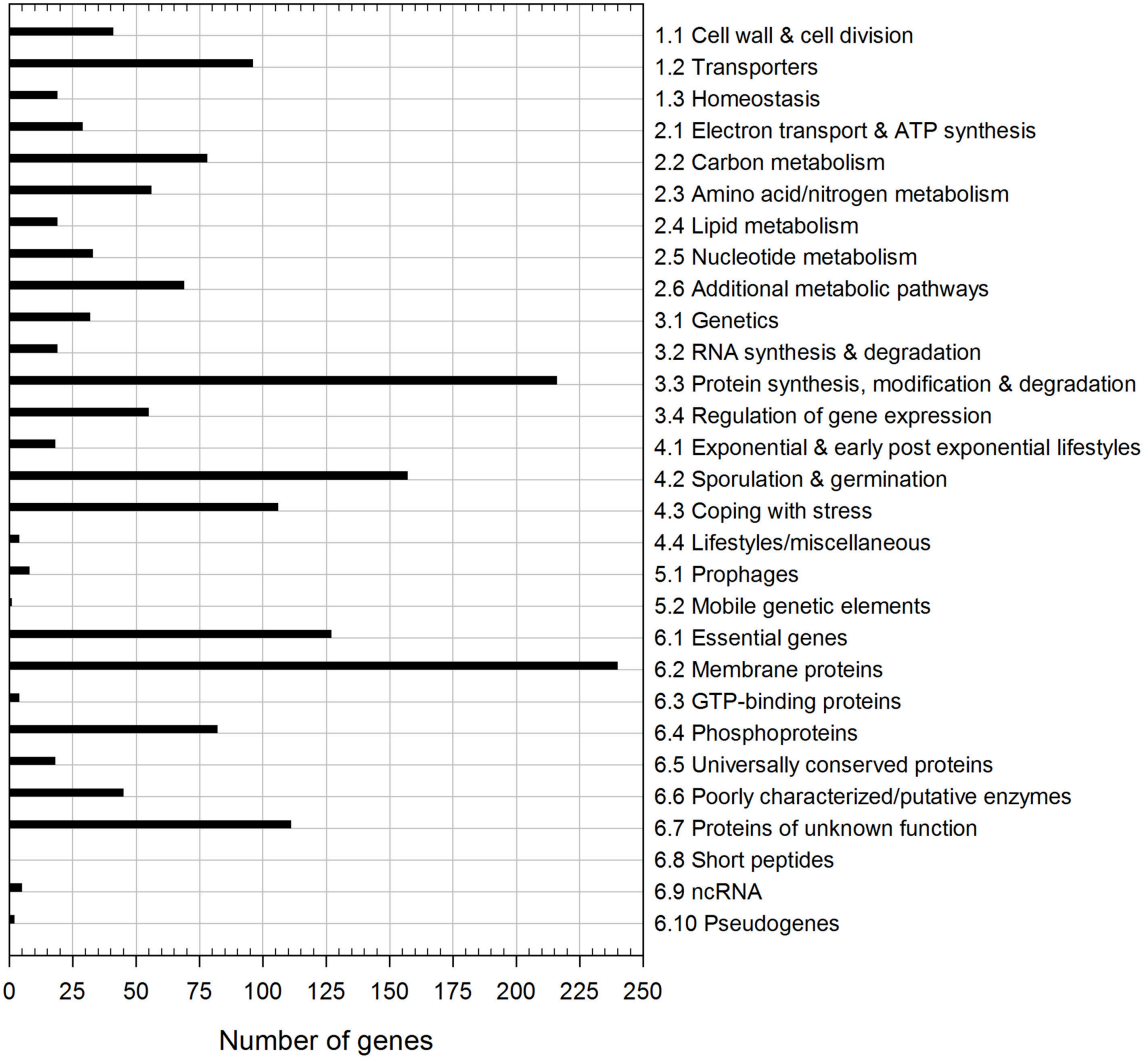
**Functional classification of dormant spore transcripts**. The 955 transcripts common to both dormant spore replicates were categorized according to *Subti*Wiki (http://subtiwiki.uni-goettingen.de).

Out of the 955 detected common dormant spore transcripts, ~25% encode membrane proteins with one-third thereof coding for transporters (Figure 2). Another prevalent group of dormant spore RNAs detected is involved in information processing (34%), especially in proteins synthesis, modification and degradation (216 transcripts). About half of these 216 transcripts were tRNA and tRNA-related genes, which had the overall highest abundances in both replicates. The tRNA reservoir may facilitate a rapid start of translation after germination is completed. Furthermore, 106 mRNAs belong to the “Coping with stress” functional category, one-third of which is a part of the σ^B^ regulon (Figure 2). If indeed translated, they may play an important role during outgrowth under suboptimal conditions. Besides, 157 dormant spore transcripts encode proteins for sporulation and germination, including the highly abundant mRNAs for small acid-soluble spore proteins (SASPs). These mRNAs are most likely residues from sporulation and might support outgrowth by providing nucleotides for *de novo* RNA synthesis (Setlow and Kornberg, 1970; Keijser et al., 2007). Finally, in agreement with previous reports (Segev et al., 2012; Bassi et al., 2016), many dormant spore RNAs (partially with very high abundance, e.g., *ytzL, yrzQ*, and *ypzG*) code for proteins of unknown function, whose characterization could yield new insights into sporulation and/or spore composition.

### Differential gene expression during outgrowth at high salinity

To investigate the impact of salt stress on the ripening phase of outgrowth, RNA was extracted 30, 60, and 90 min after the initiation of germination in the absence and presence of 1.2 M NaCl. The RNA was subjected to RNA-seq and the results were evaluated using the T-REx analysis pipeline (De Jong et al., 2015). T-REx offers two different significance thresholds, i.e., “TopHits” (log_2_FC ≥ 2, *p* ≤ 0.05) and “HighFold” (log_2_FC ≥ 5, *p* ≤ 0.01), but unless noted otherwise, TopHits-values are shown (in the following paragraph, the respective HighFold-values are given in parentheses). In all cases, the RNA-seq data obtained from outgrowth in the presence of NaCl was contrasted against the data from outgrowth in the absence of NaCl.

In total, 402 (85) genes were upregulated and 632 (190) genes were downregulated during outgrowth in the presence of 1.2 M NaCl (Table 1). At all investigated time points, the transcriptomes of dormant spores, salt-stressed outgrowing spores, and non-salt-stressed outgrowing spores were clearly distinct from each other according to principal component analysis (Figure S2). The strongest alteration of gene expression caused by the presence of NaCl was detected at 30 min of outgrowth (Table 1; Figure S2). This may be due to major salt stress response and adaptation processes occurring at this time point. In addition, high salinity might have postponed the molecular reorganization processes of early outgrowth in a similar manner as it causes a reduced growth rate of vegetative cells (Boch et al., 1994; Hahne et al., 2010). In any case, the transcriptomes of stressed vs. non-stressed outgrowing spores became more similar over time, as indicated by the lower number of differentially expressed genes in the 60 and 90 min samples (Table 1). Consistently, the 30 min transcriptome contained much more genes that were exclusively differentially expressed at this time point than the 60 and 90 min transcriptomes (Table 2). Yet, 134 (76) genes were differentially expressed at all three time points of outgrowth under salt stress (Table 2).

**Table 1:** **Differentially expressed genes during outgrowth in the presence of 1.2 M NaCl^a^**.

**Time (min)^b^**	**TopHits log_2_FC ≥ 2; *p* ≤ 0.05**		**HighFold log_2_FC ≥ 5; *p* ≤ 0.01**	
	**Upregulated**	**Downregulated**	**Upregulated**	**Downregulated**
30	321	523	64	153
60	157	184	41	25
90	118	161	31	38
Total	402	632	85	190

a *Gene expression in the presence of 1.2 M NaCl was contrasted against gene expression in the absence of NaCl at each respective time point*.

b *Time of sample withdrawal after mixing spores with germinants*.

**Table 2:** **Cohesion of contrasts: specific and shared differentially expressed genes among the sample time points**.

**Sample time point(s)**			**Number of genes^a^**	
**30 min**	**60 min**	**90 min**	**TopHits**	**HighFold**
			564	329
			90	54
			68	44
			93	42
			24	15
			53	30
			134	76

a *The numbers of genes are either specific to the time point (only one gray-filled box), or shared exclusively by the time points indicated by gray fills*.

**Figure 3:**
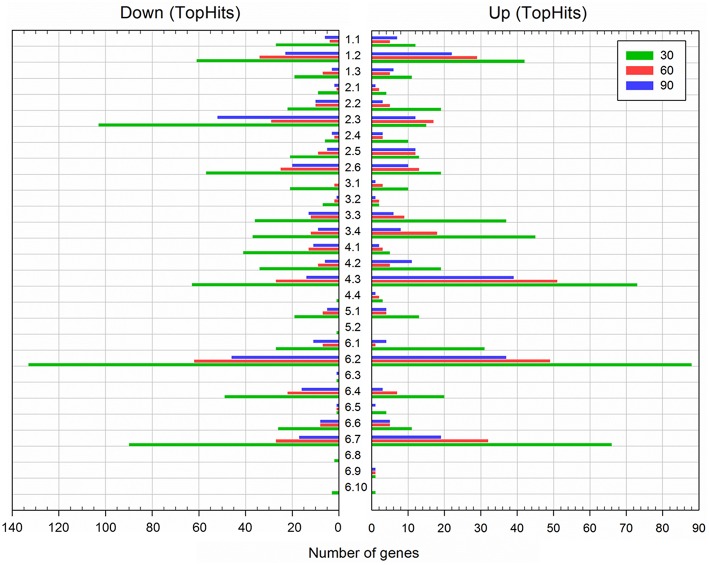
**Functional classification of differentially expressed genes during spore outgrowth in the presence of 1.2 M NaCl (TopHits)**. Genes were categorized according to *Subti*Wiki (http://subtiwiki.uni-goettingen.de). Sample time points: 30 min (green), 60 min (red), and 90 min (blue).

For functional interpretation, differentially expressed genes were categorized according to the *Subti*Wiki database (Mäder et al., 2012; Michna et al., 2016). Strong alterations of gene expression—at all-time points and in both directions—could be observed in the functional categories “Membrane proteins” (category number 6.2, see Figure 3), “Transporters” (1.2), “Coping with stress” (4.3), and “Proteins of unknown function” (6.7) (Figure 3; Tables S1, S2; Database S1). To a lesser extent and predominantly after 30 min outgrowth, genes belonging to the functional categories “Protein synthesis, modification and degradation” (3.3), “Regulation of gene expression” (3.4), and “Essential genes” (6.1) were also notably differentially expressed (Figure 3; Tables S1, S2; Database S1). While most enriched categories exhibited similar extents of up- and downregulation, genes in the categories “Amino acid/nitrogen metabolism” (2.3), “Additional metabolic pathways” (2.6), and “Phosphoproteins” (6.4) were greatly downregulated (Figure 3; Tables S1, S2; Database S1). This might relate to the aforementioned potential high-salinity-dependent retardation of the molecular reorganization processes during the ripening period and/or the reallocation of cellular resources toward salt stress response. The most relevant functional categories and groups are discussed in detail below.

### Hyperosmotic stress response

**Figure 4:**
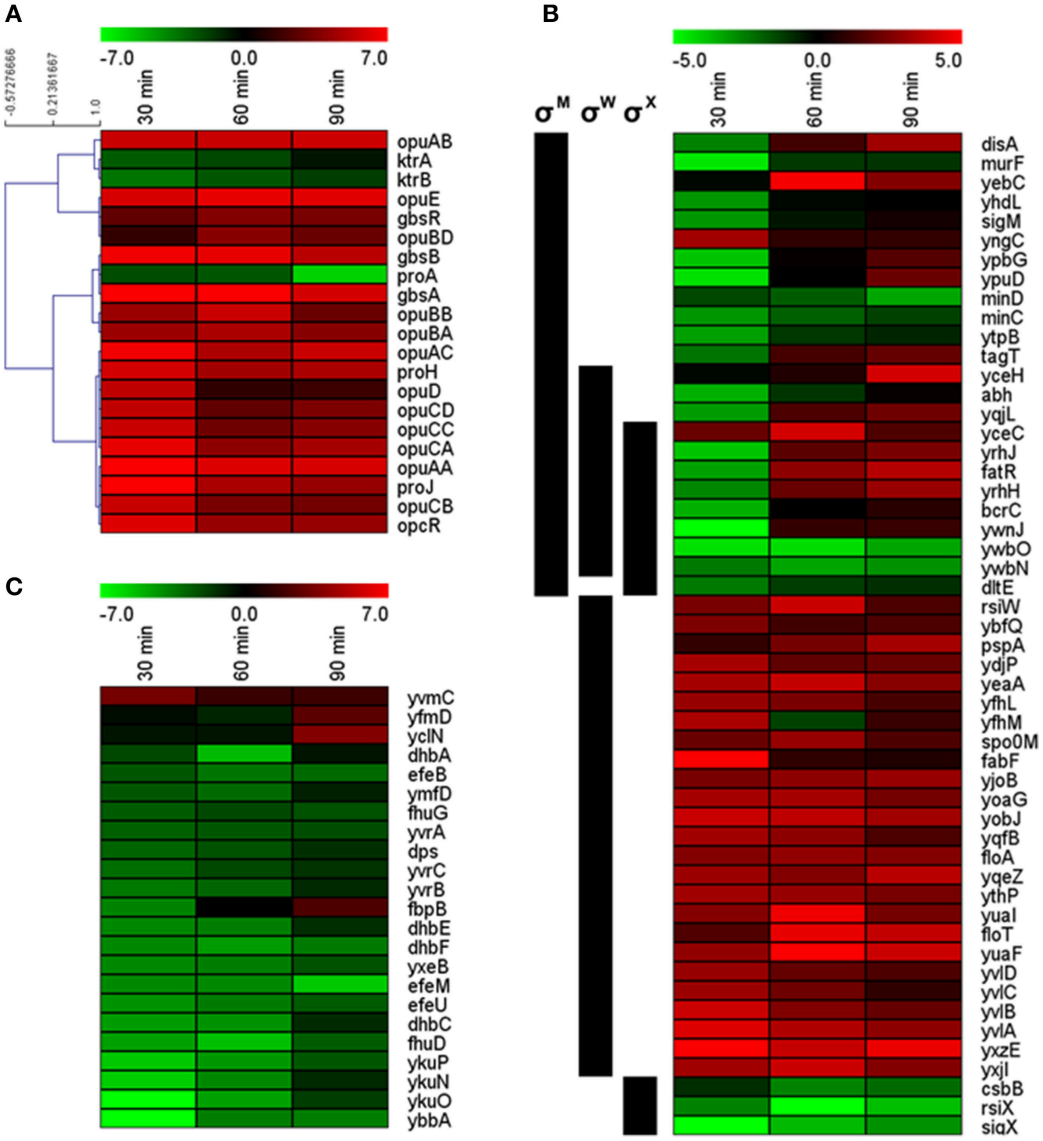
**Expression profiles of (A) genes associated with the hyperosmotic stress response, (B) differentially expressed members of the σ^M^, σ^W^, and σ^X^ regulons (regulon affiliations are indicated by black bars on the left), and (C) genes associated with iron homeostasis**. Only significantly differentially expressed genes are shown. Cutoff-values (log_2_FC) of the color scale are indicated at the top of each figure.

The functional category “Coping with hyperosmotic stress” includes genes encoding proteins involved in the specific hyperosmotic stress response, i.e., all Opu transporters (OpuA–OpuE); the K^+^ uptake systems KtrAB and KtrCD; GbsA, GbsB, and GbsR required for GB synthesis from the precursor choline; ProA, ProH, and ProJ responsible for osmoadaptive proline synthesis; and the amino-peptidases PapA and PapB that can degrade proline-containing peptides (Boch et al., 1994, 1996; Kempf and Bremer, 1998; Holtmann et al., 2003; Brill et al., 2011a; Zaprasis et al., 2013). In our study, 20 of the 25 genes in this category were differentially expressed (Figure 4A). No significant differential expression was detected for *ktrC, ktrD, papA, papB*, and *opuBC* (Dataset S1). Seventeen of the twenty differentially expressed genes were upregulated, including the operons *proHJ, gbsAB, opuA, opuB* (except *opuBC*), *opuC*, and the genes *gbsR, opuD*, and *opuE* (Figure 4A). All of these genes encode proteins involved in uptake and synthesis of osmoprotectants, which play a central role in the hyperosmotic stress response of vegetative *B. subtilis* cells (Kempf and Bremer, 1998; Bremer, 2002). Our results indicate that the same genes have an important function in the salt stress response of outgrowing spores as well.

In agreement with previous reports, salt-stressed induced upregulation of *opu* genes was already very strong after 30 min of outgrowth (Figure 4A) and was independent of the transporters' substrate availability (as no substrates were in the medium), reflecting their osmotic control (Hahne et al., 2010). It should be noted that *opuBC* from the *opuB* operon was likewise upregulated (around 2.5 log_2_FC; Dataset S1), but the difference may not have been significant as *opu* genes are reportedly also expressed to some extent in non-stressed outgrowing spores (Keijser et al., 2007). Interestingly, the different *opu* operons had variable temporal expression patterns (Figure 4A): *opuD* was only significantly upregulated at 30 min and expression of *opuC* also peaked at 30 min and was only moderately upregulated later. In contrast, *opuA* and *opuE* exhibited continuous high expression at all-time points. With regard to previous studies, the observed differential temporal expression patterns could be interpreted as follows.

During vegetative growth, *opuA* expression is elaborately balanced with the extent of the cell's internal solute pool, as sufficient intracellular amounts of osmoprotectants repress *opuA* transcription (Hoffmann et al., 2013). Thus, steady upregulation of the *opuA* operon during outgrowth at high salinity indicates that the outgrowing spores were not able to accumulate sufficient amounts of compatible solutes throughout the whole experiment (Figure 4A). Moreover, steady expression of *opuE*, which has a σ^A^- as well as a σ^B^-dependent promoter, is mediated by σ^A^ during sustained salt stress (Spiegelhalter and Bremer, 1998). Therefore, the observed steady upregulation of *opuE* in our study suggests that the osmospecific stress response governed by σ^A^ is important for the salt stress adaptation during outgrowth. While the early, transient upregulation of *opuD*, which is also controlled by both σ^A^ and σ^B^ promoters, would rather resemble σ^B^-dependent transcription, a similar pattern was detected for the *opuC* operon that is not a member of the σ^B^-regulon (Spiegelhalter and Bremer, 1998; Hoffmann and Bremer, 2011; Young et al., 2013). At least in part, the transient upregulation of *opuD* and the *opuC* operon was due to an incremental transcription in non-stressed outgrowing spores (GSE81238). Furthermore, *opuC* transcription might have been affected by its repressor *opcR*: although the biological function of this *gbsR*-type repressor is still unknown (Lee et al., 2013), its expression pattern was strikingly similar to that of the *opuC* operon (Figure 4A).

Compared to the other *opu* genes, upregulation of the *opuB* genes was more variable; the overall expression levels, however, were comparably low (Figure 4A; GSE81238). Nevertheless, *opuB* transcription may likewise have been modulated by the repressor GbsR, which regulates choline uptake (via OpuB) and processing to GB (via GbsA and GbsB), and expression of which was upregulated as well during outgrowth at high salinity (Figure 4A; Nau-Wagner et al., 2012). GbsR can directly bind choline, which leads to derepression of the *opuB* and *gbsAB* operons, allowing efficient accumulation of GB (Boch et al., 1996; Nau-Wagner et al., 2012). Strikingly, despite *gbsR* upregulation and the absence of choline, *gbsAB* was upregulated in our experiment. The reason for this *gbsAB* upregulation remains to be determined.

Further evidence for the importance of compatible solutes during outgrowth under salt stress was the strong upregulation of the osmoadaptive proline synthesis genes *proH* and *proJ*. In vegetative cells, osmotic induction of the *proHJ* operon can be observed after a salt shock as well as during sustained high salinity and is mediated by an osmotically controlled σ^A^-type promoter (Steil et al., 2003; Hahne et al., 2010; Brill et al., 2011a). Although osmoadaptive proline synthesis requires ProA (encoded in the not osmotically inducible *proBA* operon), *proA* was found to be repressed during outgrowth in high salt conditions (Figure 4A), which is in agreement with previous findings (Hahne et al., 2010; Brill et al., 2011a). Transcription of the *proBA* operon is regulated by a tRNA-responsive riboswitch, allowing *proBA* derepression only upon proline starvation (Brill et al., 2011b). Thus, the downregulation of *proA* in salt-stressed outgrowing spores may in fact signify derepression of *proBA* in non-stressed outgrowing spores, as these likely have a higher anabolic proline turnover due to a higher protein biosynthesis rate.

While most genes involved in the accumulation of osmoprotectants were upregulated during outgrowth at high salinity, the not osmotically inducible *ktrAB* operon was found to be downregulated at all-time points (Figure 4A). Since the KtrAB transporter system plays an important role in K^+^ uptake as a first defense against high osmolality (Holtmann et al., 2003) it is possible that the K^+^ accumulation phase had already ended within the first 30 min of outgrowth and *ktrAB* expression was downregulated at ≥30 min to prevent detrimental effects of further K^+^ uptake. As *ktrAB* is regulated by *ydaO*-type riboswitch causing increased transcription termination in the presence of c-di-AMP (Nelson et al., 2013) and transcript levels of *ktrAB* were very low in salt-stressed outgrowing spores (GSE81238), it would be intriguing to investigate the role of c-di-AMP signaling in salt stress responses and outgrowth in more detail in future studies.

### General stress response

Numerous previous studies on osmotically stressed vegetative cells of *B. subtilis* have indicated an induction of the σ^B^-dependent general stress response upon sudden osmotic increases (e.g., Spiegelhalter and Bremer, 1998; Petersohn et al., 2001; Höper et al., 2006; Hecker et al., 2007; Nannapaneni et al., 2012; Nicolas et al., 2012; Young et al., 2013). As spores germinated in high-salinity media were also suddenly confronted with salt stress, an involvement of the general stress response would seem plausible. The *sigB* gene itself was not differentially expressed at any sample time point (Dataset S1), which was not surprising as σ^B^ is only transiently active after a salt shock (Spiegelhalter and Bremer, 1998; Young et al., 2013). Yet, in total, almost one-third of the σ^B^ regulon was differentially expressed at one or several sample time points: 14 genes were significantly upregulated, whereas 31 genes were downregulated (Table 3; Figure S3A). Importantly, all upregulated genes except for the uncharacterized *ydeC* have additional regulators aside from σ^B^ (e.g., σ^W^), which were likely responsible for the increased expression (see below). Moreover, only one of the 37 general stress response genes whose absence causes a salt-sensitive phenotype (i.e., *yflH;* Höper et al., 2005) was upregulated, whereas seven others were downregulated (Figure S3A). Indeed, σ^B^ was previously reported to be dispensable for colony formation from spores at high salinity (Tovar-Rojo et al., 2003). Altogether, our data suggest that the σ^B^-dependent general stress response is not of major significance during outgrowth in the presence of 1.2 M NaCl.

**Table 3:** **Involvement of alternative sigma factors in the salt stress response of outgrowing spores^a^**.

**Regulon**	**Description**	**Number of genes^b^**	**Differentially expressed^c^**		
			**%**	**#up**	**#down**
*sigB*	General stress response	151	30	14	31
*sigD*	Regulation of flagella, motility, chemotaxis, and autolysis	24	33	0	27
*sigE*	Sporulation (early mother cell-specific)	176	9	8	7
*sigF*	Sporulation (early forespore-specific)	63	11	2	5
*sigG*	Sporulation (late forespore-specific)	108	10	3	8
*sigH*	Transcription of early stationary phase genes (sporulation, competence)	37	43	1	14
*sigI*	Control of a class of heat shock genes	6	50	0	3
*sigK*	Sporulation (late mother cell-specific)	103	7	2	4
*sigL*	Utilization of arginine, acetoin, and fructose; required for cold adaptation	23	52	0	12
*sigM*	ECF-type sigma factor responsible for intrinsic resistance against beta-lactam antibiotics	69	35	4	13
*sigO-rsoA*	Two-subunit sigma factor	5	20	1	0
*sigV*	ECF-type sigma factor; response to lysozyme	4	0	0	0
*sigW*	ECF-type sigma factor; activated by alkaline shock, polymyxin B, vancomycin, cephalosporin C, D-cycloserine, and triton X-100	65	54	28	4
*sigX*	ECF-type sigma factor; cell surface properties	29	45	1	9
*sigY*	ECF-type sigma factor; maintenance of the SPβ prophage	7	0	0	0
*ylaC*	ECF-type sigma factor; response to oxidative stress	4	0	0	0
*xpf*	PBSX phage RNA polymerase sigma factor	10	50	0	5

a *Classification and description according to SubtiWiki and (Souza et al., 2014)*.

b *Number of genes within the regulon*.

c *The numbers of up- (#up) and downregulated (#down) genes only include genes that have the same expression direction (up/downregulated) at all three time points. The percentage of differentially expressed genes includes all genes*.

### Sigma factors and regulons

Aside from σ^B^, the alternative sigma factors σ^M^, σ^W^, and σ^X^ have repeatedly been implicated with cell envelope and salt stress (Horsburgh et al., 2001; Petersohn et al., 2001; Steil et al., 2003; Höper et al., 2006; Hahne et al., 2010). Hence, we analyzed the role of the different alternative sigma factors and major regulons in the salt stress response of outgrowing spores.

Among the genes encoding alternative sigma factors, only three significant changes were observed: downregulation of *sigM* at 30 min outgrowth, upregulation of *sigO* at 60 min outgrowth, and downregulation of *sigX* throughout the whole experiment (Dataset S1). It should be noted that upregulation of *sigO*, which forms a two-subunit sigma factor with RsoA, is almost certainly an artifact, because this gene was barely expressed at all and *rsoA* was not differentially expressed (GSE81238). While the repression of *sigX* under salt stress has previously been reported (Steil et al., 2003; Hahne et al., 2010; Nicolas et al., 2012), the lack of differential *sigW* expression was in contrast to the study by Hahne et al. (2010), in which a significant *sigW* upregulation 30 and 60 min after the osmotic upshift has been shown.

Nevertheless, more than half of the genes in the σ^W^ regulon were differentially expressed, most of them being upregulated (Table 3; Figure 4B), indicating importance of σ^W^ for the salt stress response during outgrowth. Interestingly, while Hahne et al. (2010) reported a maximum induction of the σ^W^ regulon at 30 min after NaCl addition, we observed three different expression patterns within this regulon: (i) 27 genes were upregulated at all-time points, (ii) four genes were downregulated at all-time points, and (iii) four genes were downregulated at 30 min and upregulated subsequently (Figure 4B). Most likely the distinct expression patterns of the latter two gene groups were caused by their simultaneous control by σ^M^ and σ^X^ (Figure 4B). The constant upregulation of the σ^W^ regulon also supports the notion that the salt stress response of outgrowing spores is rather similar to that of cells growing at sustained high salinity and not to that of salt-shocked cells, as salt-shocked cells shut down the σ^W^ regulon about 20 min after the osmotic upshift (Steil et al., 2003).

Additionally, one-third of the σ^M^ regulon was differentially expressed, although 10 out of these 24 genes were also members of the σ^W^ regulon (Table 3; Figure 4B). While Hahne et al. (2010) have reported maximum upregulation of the σ^M^ regulon 60 min after NaCl addition, σ^M^-dependent genes tended to be repressed in our study (Figure 4B). Downregulation was predominant at 30 min of outgrowth, which is consistent with the repression of the *sigM* gene at this time point. However, at the later time points, most genes became less repressed or were even upregulated (Figure 4B). Altogether, an involvement of σ^M^ in salt stress adaptation during outgrowth is possible, but the tendency for downregulation—in context with the salt-sensitive phenotype of *sigM* mutants (Horsburgh et al., 2001)—suggests that σ^M^-dependent genes may be dispensable during the early outgrowth phase under salt stress. In consistence with previous reports (Steil et al., 2003; Hahne et al., 2010), the σ^X^ regulon was largely repressed during outgrowth at high salinity (Table 3). Notably, all differentially expressed σ^X^ genes (except *sigX* and the anti-σ^X^ factor *rsiX*) were also members of the σ^W^ and/or σ^M^ regulon (Figure 4B).

As summarized in Table 3, various genes belonging to the regulons of other alternative sigma factors were differentially expressed during outgrowth at high salinity as well. Most notably, many σ^D^–dependent genes involved in motility and chemotaxis were repressed throughout the entire experiment (Figure S3B), which is in excellent agreement with previous findings from salt-stressed vegetative cells (Steil et al., 2003; Höper et al., 2006; Hahne et al., 2010; Nicolas et al., 2012). Furthermore, all differentially expressed genes of the σ^I^, σ^L^, and Xpf regulons were downregulated in our experiment (Table 3; Dataset S1). Some differential expression was also detected within the sporulation-related σ^E^, σ^F^, σ^G^, σ^K^, and σ^H^ regulons (Table 3). However, the portion of differential expression within the σ^E^, σ^F^, σ^G^, and σ^K^ regulons was relatively low and can be explained by very low expression levels, non-sporulation-related gene functions, and/or different degradation rate of dormant spore transcripts in stressed vs. non-stressed outgrowing spores (Dataset S1). All differentially expressed σ^H^-genes except the σ^W^-dependent *spo0M* were downregulated (Table 3), consistent with the role of σ^H^ in sporulation initiation that is blocked at high salinity (Ruzal et al., 1998; Widderich et al., 2016).

Alignment of all *Subti*Wiki-annotated regulons with our data exhibited additional overlaps. While some regulators seemed to be active during outgrowth at high salinity (e.g., BirA, PyrR, LutR), genes in many other regulons behaved exactly opposite to their regulators' functions as repressors or activators (e.g., AzlB, PucR, RocR, Zur; Table S3). The latter observation suggests that these regulons were actively regulated in the non-stressed outgrowing spores. Consistently, *azlB* and *zur* have been reported to be overexpressed during outgrowth under non-stress conditions (Keijser et al., 2007).

While the DegS/DegU two-component system has previously been implicated in salt stress sensing and response (Ruzal and Sánchez-Rivas, 1998; Mäder et al., 2002; Steil et al., 2003), only five genes (19%) of the DegU regulon were differentially expressed in our study (Table S3; Dataset S1). Moreover, there was only a limited overlap (38%, mostly motility genes) with the DegS/DegU regulated genes that were reported to be differentially expressed in salt-stressed vegetative cells (Steil et al., 2003). Perhaps, the lack of major changes in the DegU regulon can be explained by the upregulation of *rapG*, which encodes a DegU-inhibiting response regulator aspartate phosphatase (Ogura et al., 2003). Nevertheless, the role of DegS/DegU in salt stress adaption during spore outgrowth remains questionable.

Induction of the PerR regulon in vegetative, salt-stressed cells has previously been hypothesized to indicate increased oxidative stress caused by high salinity (Höper et al., 2006). However, in our study, the PerR-dependent catalase gene *katA* was repressed while the other members of the regulon were not differentially expressed (Table S3; Dataset S1).

### Overlap with other stress responses

As many survival strategies of *B. subtilis* are closely interlinked (Höper et al., 2005; Lopez et al., 2009; Schultz et al., 2009), the transcriptional profile of genes known to be involved in other stress responses was analyzed as well (summarized in Table S4). Our data indicate a large overlap (about 40%) of differential gene expression during outgrowth at high salinity and cell envelope stress, largely reflecting the changes in the σ^M^, σ^W^, and σ^X^ regulons described above (Table S4; Figure 4B). Notably, more than half of the 35 cell envelope stress-related genes that exhibited significant upregulation at one or more sample time points encoded hypothetical and poorly characterized proteins, whose role may be interesting to investigate in the future (Dataset S1). In addition, several genes encoding heat shock proteins, chaperones (e.g., *groEL, groES, dnaK*), and proteases (e.g., *clpE, clpX*) were upregulated in our experiment (Dataset S1). Protein quality control is important during outgrowth (Sinai et al., 2015) and likely even more so during outgrowth at high salinity, since osmotic upshifts have been proposed to cause protein denaturation and misfolding (Hahne et al., 2010). Additional overlaps between our data and genes involved in other stress responses are of unknown functional relevance and include the categories “Resistance against toxins/antibiotics” (27% overlap, 17 upregulated, 11 downregulated), “Resistance against oxidative and electrophile stress” (22% overlap, four upregulated, nine downregulated), and “Biosynthesis of antibacterial compounds” (27% overlap, four upregulated, 11 downregulated; Table S4; Dataset S1).

### Cell envelope

**Figure 5:**
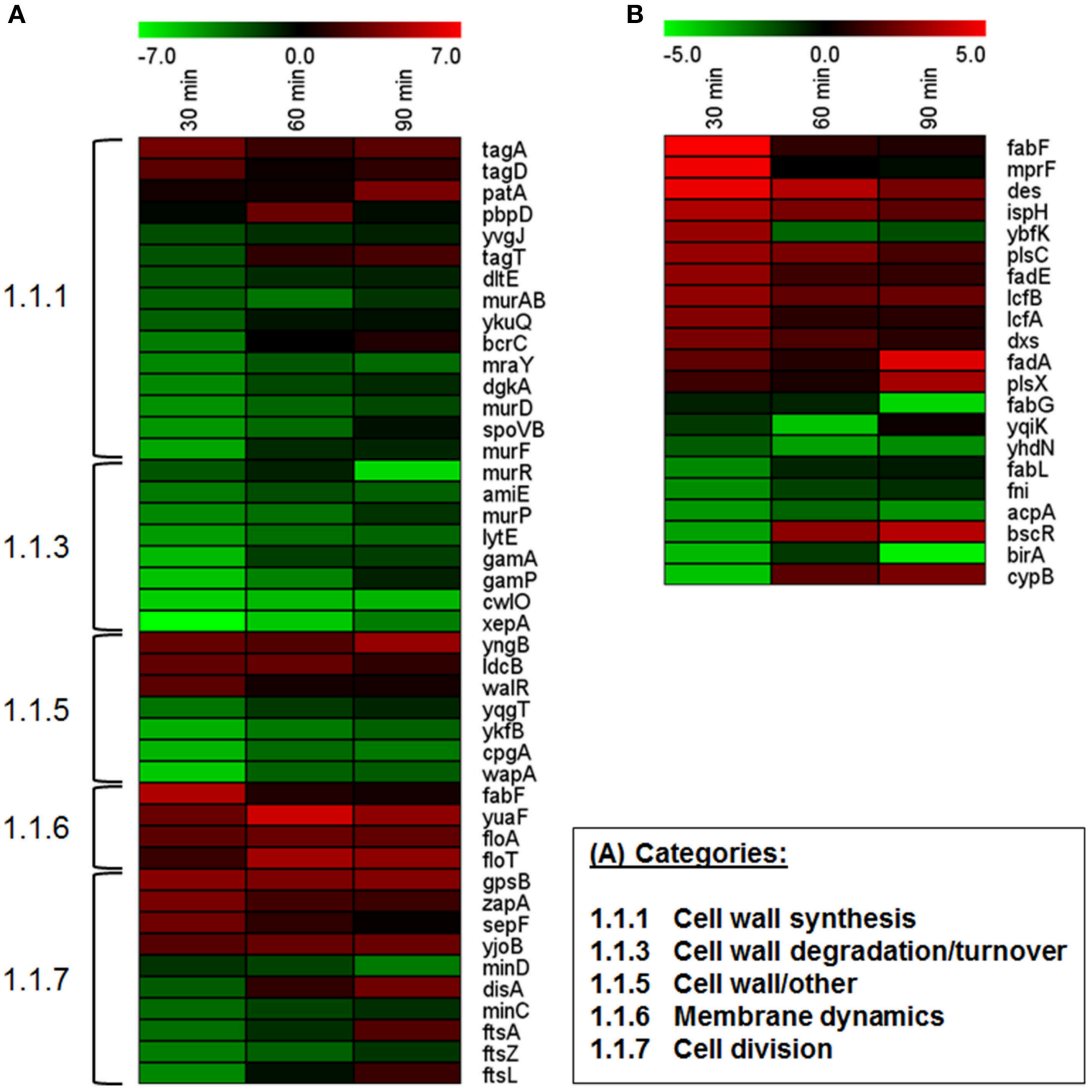
**Expression profiles of (A) genes associated with cell wall and cell division and (B) genes involved in lipid metabolism**. Only significantly differentially expressed genes are shown. Cutoff-values (log_2_FC) of the color scale are indicated at the top of each figure.

Vegetative *B. subtilis* cells growing under hyperosmotic conditions exhibit alterations of their cell envelope, i.e., changes in cell wall structure and membrane composition (López et al., 1998, 2006; Palomino et al., 2009; Hahne et al., 2010). During outgrowth at high salinity, cell envelope stress also seems to be apparent (see above). In total, 44 genes (ca. 23%) of the category “Cell wall” were differentially expressed (Dataset S1). Among these, especially the genes involved in cell wall turnover (8 genes) and cell wall synthesis (10 genes) were repressed, although *tagA* and *tagD* involved in early steps of teichoic acid biosynthesis were upregulated (Figure 5A). PBP4^*^ (encoded by *pbpE*) has previously been proposed to play a role in peptidoglycan modification at high salinity (Palomino et al., 2009; Hahne et al., 2010), but the gene was not differentially expressed in our study (Dataset S1).

High-salinity-induced changes in the membrane composition of vegetative cells include an increase in saturated straight-chain fatty acids, unsaturated fatty acids, and cardiolipin, and a decrease in branched fatty acids (López et al., 1998, 2006). During outgrowth at high salinity, 21 out of 109 genes involved in lipid metabolism were differentially expressed (Dataset S1). Genes encoding enzymes for fatty acid utilization tended to be upregulated (Figure 5B), consistent with a previously reported global upregulation of genes involved in degradation of free fatty acids via β-oxidation (Hahne et al., 2010). In contrast, genes important for fatty acid and lipid biosynthesis exhibited expression changes in both directions. Since only little is known about membrane remodeling during spore outgrowth, the relevance of the observed transcriptomic differences and their actual impact on membrane composition is unclear. Nevertheless, in agreement with a possible increase in unsaturated fatty acids (López et al., 1998), the *des* gene encoding a fatty acid desaturase was upregulated (Figure 5B). Interestingly, although anionic phospholipids (in particular cardiolipin) play a role in osmoadaptation, possibly by changing biophysical membrane properties such as fluidity (Poolman et al., 2004; López et al., 2006; Romantsov et al., 2009; Unsay et al., 2013), neither the genes encoding cardiolipin synthases (*clsA, ywiE, ywjE*) nor *pgsA* (encoding a phosphatidylglycerophosphate synthase) were differentially expressed (Dataset S1). However, σ^W^-dependent upregulation of *fabF* as well as *yuaF* and the flotillin-homologs *floT* and *floA* (Figure 4B, Dataset S1) suggests that changes of the cytoplasmic membrane (e.g., *fabF*-induced fluidity decrease) may play a role in osmoadaptation of outgrowing spores (Kingston et al., 2011).

### Membrane proteins and transporters

The membrane protein and transporter transcriptome of outgrowing spores was severely altered by the presence of 1.2 M NaCl (Figure 3). In total, 54 ABC transporter genes (i.e., 26% of genes in this category), six phosphotransferase system genes (21%), and 65 other transporter genes (33%) were differentially expressed (Dataset S1). With regard to the importance of solute pool adjustments during osmotic stress adaptation on the one hand, and major molecular reorganization during outgrowth on the other hand, this is not surprising and in agreement with previous transcriptomic and proteomic studies (Steil et al., 2003; Keijser et al., 2007; Hahne et al., 2010; Segev et al., 2013; Hoffmann and Bremer, 2016).

Overall, 44% of differentially expressed transporter genes exhibited upregulation (Dataset S1) and included (aside from *opu* transporter genes) many genes encoding cation efflux transporters: the *khtSTU* operon involved in K^+^ efflux, *mrpABC* of the *mrpABCDEFG* operon encoding *B. subtilis*' major Na^+^ extrusion system, as well as *nhaC* and *nhaK* involved in Na^+^ and monovalent cation efflux, respectively (Fujisawa et al., 2004, 2005; Gorecki et al., 2014). Upregulation of K^+^ efflux systems, which likewise occurs in salt-stressed cells (Steil et al., 2003; Hahne et al., 2010), is in agreement with the observed downregulation of the KtrAB K^+^ importer (see above; Figure 4A). Both transcriptional changes might constitute consequences of the initial K^+^ uptake phase of osmoadaptation (Whatmore et al., 1990) that may have ended before 30 min into outgrowth. Transcriptomic changes toward increased Na^+^ efflux most likely represent countermeasures against high intracellular Na^+^ concentrations that are toxic for *B. subtilis*, but may for instance build up due to the Na^+^-coupled activity of the OpuD and OpuE transporters (Figure 4A; Gorecki et al., 2014; Hoffmann and Bremer, 2016). This would not only be in agreement with observations from vegetative cells (Hahne et al., 2010), but also with the downregulation of Na^+^ symporter genes (*yocR, yrbD, yocS, putP, yodF*) in our study (Dataset S1).

Further upregulated transporter genes are functionally involved (i) in the uptake of zinc, phosphate, sulfonate, sulfate, glucose, gluconate, uracil, and cysteine; (ii) in branched-chain amino acid transport; and (iii) in the export of toxic peptides and antibiotics (Dataset S1). Although we detected upregulation of two genes for iron uptake (only at 90 min) and *sufC* involved in the synthesis of Fe-S clusters that were implicated in the salt stress response of vegetative cells (Höper et al., 2006), unexpectedly many iron and iron siderophore uptake systems were downregulated (see below; Figure 4C). While the di- and tripeptide importer *dtpT*, which can contribute to osmoprotection by taking up proline-containing peptides, was upregulated, genes encoding Dpp and Opp implicated in the same osmoprotective function (Zaprasis et al., 2013) were downregulated (Dataset S1). As it is possible that non-stressed outgrowing spores utilize these uptake systems to gain access to a broader nutrient spectrum, the relevance of extracellular peptides in outgrowth and salt-stress adaptation of outgrowing spores remains to be determined. Generally it is plausible that downregulation of many transporter genes in our study was in fact a consequence of higher metabolic and/or biosynthetic activity in non-stressed cells, as these genes are involved in the uptake of common metabolites including purines, nitrate, lactate, and various amino acids (Dataset S1).

In total, transporter genes constituted 42% of the differentially expressed membrane protein genes, emphasizing the importance of transport processes during outgrowth and osmoadaptation within this phase (Figure 3; Table S1). The functions of the membrane proteins encoded by the residual 58% of differentially expressed genes in this category were very diverse and included genes for regulatory proteins, flagellum, and chemotaxis, kinases, dehydrogenases, cell division proteins, and many poorly characterized proteins (Dataset S1). Moreover, about 30% of the differentially expressed non-transporter membrane protein genes encoded hypothetical proteins, some of which may be interesting to investigate in more detail with regard to their role in outgrowth and osmoadaptation.

### Iron homeostasis

Iron homeostasis is governed by the central iron regulatory protein Fur, which upon binding of excess iron becomes an active repressor (Hoffmann et al., 2002; Helmann, 2014). Previous studies showed that vegetative *B. subtilis* cells grown in SMM containing 1.2 M NaCl experience iron limitation, with genes involved in the synthesis of the iron siderophore bacillibactin (*dhbACEBF* operon) and other members of the Fur regulon becoming derepressed (Hoffmann et al., 2002; Steil et al., 2003). In contrast, in our study, *dhbA, dhbC, dhbE*, and *dhbF* and 15 other genes of the category “Acquisition of iron” (1.3.3) were repressed in the presence of 1.2 M NaCl (Figure 4C; Dataset S1). Although the downregulation of only 17 genes (34%) of the Fur regulon was significant, all residual genes in this regulon were also downregulated (albeit below the significance threshold), possibly due to *fur* overexpression during outgrowth under non-stress conditions (Keijser et al., 2007). Overall, the downregulation of the Fur regulon in salt-stressed outgrowing spores may indicate a lower iron requirement, sparing a larger amount of iron to keep Fur active and its regulon repressed, thereby leading to lower iron acquisition. Possibly, the iron depot of spores of around 40 μg Fe/g (dry weight) (Granger et al., 2011) is sufficient for the potentially slower spore ripening processes at high salinity.

### Metabolism

As the outgrowing spores in our study simultaneously had to cope with molecular rearrangements for metabolic initiation and osmoadaptation, it is not surprising that 372 genes involved in metabolism were differentially expressed. High salinity affected all aspects of metabolism, but various processes were influenced to different extents: genes in the categories “Electron transport and ATP synthesis” (2.1) and “Lipid metabolism” (2.4) were affected the least; genes of “Carbon metabolism” (2.2) and “Nucleotide metabolism” (2.5) exhibited intermediate alterations; and genes involved in “Amino acid/nitrogen metabolism” (2.3) and “Additional metabolic pathways” (2.6) were affected the most (Figure 3; Tables S1, S2).

In total, 70% of the differentially expressed metabolic genes were downregulated, reflecting the detrimental effects of salt stress and suggesting a metabolic decline comparable to the reduced growth rate or growth arrest that can be observed in vegetative salt-stressed cells (Boch et al., 1994; Hahne et al., 2010). Especially the genes related to amino acid metabolism (i.e., biosynthesis, acquisition, and utilization) experienced strong repression (Figure 3; Dataset S1), which is likely to result in generally slower adaptation and ripening processes, both requiring protein biosynthesis (Segev et al., 2013; Sinai et al., 2015; Hoffmann and Bremer, 2016). Moreover, global downregulation of the amino acid metabolism may further impede synthesis of osmoprotective proline from other amino acid precursors (Zaprasis et al., 2015). Although the acquisition of adequate osmoprotectant pools seems *per se* unlikely given the extremely nutrient-poor conditions of our experiment, amino acids that the outgrowing spores may acquire from SASP degradation or from peptides liberated from the spore coat seem to have low chances to be converted to proline based on our transcriptomic data (Tovar-Rojo et al., 2003; Zaprasis et al., 2013).

Despite the apparently restrained metabolism during outgrowth at high salinity, only 12 genes involved in carbon core metabolism were downregulated in our study (Dataset S1). Previous studies on salt-stressed vegetative cells indicated that the enzymes of the tricarboxylic acid cycle canalized toward 2-oxoglutarate synthesis and thus ultimately toward glutamate and subsequent proline synthesis (Höper et al., 2006; Hahne et al., 2010). However, we could not detect such an adaptation in our experiment, as most of the tricarboxylic acid cycle genes were either not differentially expressed or even downregulated (Dataset S1).

Next to numerous downregulated metabolic routes, the only pathway that was uniformly, significantly upregulated was the uridine-5-phosphate synthesis pathway, with all eight *pyr* genes (*pyrABCDEFK*) involved in conversion of hydrogen carbonate to UMP showing strong upregulation (average log_2_FC = 7.6) at all investigated time points (Dataset S1). In contrast, six out of seven differentially expressed purine biosynthesis and acquisition genes as well as all 12 nucleotide utilization genes were downregulated. Unfortunately, the relevance of this difference in pyrimidine and purine assimilation is not clear.

Taken together, our data indicate that high salinity exerted manifold detrimental effects on the metabolism of outgrowing spores, which are especially grave given the low nutrient availability in our outgrowth medium.

## Conclusions

In its natural habitats, *B. subtilis* is frequently exposed to increases in environmental salinity, which has profound influences on cellular physiology and triggers adaptive responses (Bremer, 2002; Hoffmann and Bremer, 2016). High salinity exerts detrimental effects on *B. subtilis* spore formation (Ruzal et al., 1998; Widderich et al., 2016), spore germination (Nagler et al., 2014, 2015, 2016; Nagler and Moeller, 2015), and, as shown here and previously, spore outgrowth (Tovar-Rojo et al., 2003; Nagler et al., 2014, 2016). Although it seems counter-intuitive that high salinity inhibits the formation of desiccation resistant spores, blocking this costly cellular differentiation program most likely reflects the inability of starving cells to gather sufficient resources (e.g., for the massive production of osmoprotective proline) required for sporulation during simultaneous salt stress (Brill et al., 2011a; Widderich et al., 2016). In contrast, spore germination and outgrowth can be initiated under non-growth-permissive salt conditions, likely resulting in a survival disadvantage and indicating the lack of a counteracting sensory and regulatory response system (Boch et al., 1994; Nagler et al., 2014, 2016).

Although we exposed spores to a severe salt shock simultaneously to the germination stimulus, the σ^B^-directed stress response system surprisingly did not seem to be of major significance during this treatment. This suggests that (i) the stressosome controlling σ^*B*^ activity subsequent to a salt shock is either not (sufficiently) present in outgrowing spores, or (ii) that the cellular signal(s) controlling the release of the alternative transcription factor σ^B^ from its anti-sigma factor RsbW cannot be generated in outgrowing spores (Hecker et al., 2007; Marles-Wright and Lewis, 2010; Young et al., 2013; Hoffmann and Bremer, 2016).

The transcriptional profile of salt-stressed outgrowing spores resembled that of *B. subtilis* cells actively growing under continuous high-salinity conditions in many aspects (Steil et al., 2003; Hahne et al., 2010). Hence, the signals that trigger adaptive responses of *B. subtilis* to counteract sustained high salinity can apparently be perceived by outgrowing spores as well. At all investigated time points, salt-stressed outgrowing spores induced their complete genetic repertoire of osmoprotectant uptake and compatible solute synthesis, emphasizing the pivotal role of these substances also during outgrowth (Figure 4A). Unfortunately, the nature of the signal allowing osmotic induction of compatible solute uptake and biosynthesis systems in *B. subtilis* remains to be determined (Bremer, 2002; Hoffmann and Bremer, 2016).

In outgrowing spores, the σ^D^ regulon was strongly downregulated in response to high salt concentrations (Figure S3), indicating that flagellar biosynthesis, assembly and swimming will be impaired in the emerging vegetative cells. This is surprising as one may have predicted that chemotaxis and the ability to swim would be useful traits to escape from osmotically unfavorable to nutritionally favorable conditions (Wong et al., 1995). Perhaps, the requirement to synthesize 20,000 flagellin subunits (Hag) for the production of a single filament is too resource-consuming for salt-stressed outgrowing spores (Mukherjee and Kearns, 2014). However, the strong down-regulation of flagellar genes and the concomitant abrogation of swimming have also been observed in *B. subtilis* cells exposed to prolonged high salinity (Steil et al., 2003).

In conclusion, our study provided new insights on the transcriptomic adaptations of outgrowing spores to the presence of high salt concentrations and points out another facet of the perturbing effects that osmotic stress can exert on the life cycle of spore-forming soil bacteria.

## Author contributions

KN designed, performed, and evaluated the transcriptomics and spectrophotometric germination experiments, and prepared the text and figures of the manuscript. AK supported experimental design, gave scientific input, and edited the manuscript text. AD supported evaluation and handling of the transcriptomic data. OK arranged RNA sequencing and edited the manuscript text. KM and ML performed and evaluated the live cell imaging and scanning electron microscopy experiments, and edited the manuscript text. EB and TH supported evaluation of transcriptomic data and edited the manuscript text. RM gave scientific input and edited the manuscript text. All authors read and approved the final manuscript.

## Funding

This work was supported by the Helmholtz Space Life Sciences Research School (SpaceLife) and the German Aerospace Center of the Helmholtz Association (Ph.D. fellowship of KN). RM and KN were supported by DLR grant DLR-FuE-Projekt ISS LIFE, Programm RF-FuW, Teilprogramm 475.

### Conflict of interest statement

The authors declare that the research was conducted in the absence of any commercial or financial relationships that could be construed as a potential conflict of interest.

## Abbreviations

2x SG: Modified Schaeffer's Sporulation Medium with glucose
DPA: Dipicolinic acid (pyridine-2,6-dicarboxylic acid)
FC: Fold change
GB: Glycine betaine
OD_600*nm*_: Optical density at 600 nm
RNA-seq: RNA sequencing
SASP: Small, acid-soluble spore protein
SEM: Scanning electron microscopy
SMM: Spizizen Minimal Medium.
