# Supplementary material for: Identification of Differentially Expressed Genes during Bacillus subtilis Spore Outgrowth in High-Salinity Environments Using RNA Sequencing
Source: Front Microbiol. 2016 Oct 6;7:1564. doi: 10.3389/fmicb.2016.01564 (PMC5052260; doi:10.3389/fmicb.2016.01564)
Supplement: Supplementary file 2 [file DataSheet2.PDF]

Supplementary Material

Identification of differentially expressed genes during *Bacillus subtilis* spore outgrowth in high-salinity environments using RNA sequencing

Katja Nagler[1], Antonina O. Krawczyk[2], Anne de Jong[2], Kazimierz Madela[3], Tamara Hoffmann[4], Michael Laue[3], Oscar P. Kuipers[2], Erhard Bremer[4], and Ralf Moeller[1]*

[1] Space Microbiology Research Group, Radiation Biology Department, Institute of Aerospace Medicine, German Aerospace Center, Cologne, Germany,

[2] Department of Molecular Genetics, Groningen Biomolecular Sciences and Biotechnology Institute, University of Groningen, Groningen, The Netherlands,

[3] Advanced Light and Electron Microscopy, Center for Biological Threats and Special Pathogens, Robert Koch Institute, Berlin, Germany,

[4] Laboratory of Microbiology, Department of Biology, Philipps-University Marburg, Marburg, Germany.

Running title: High-salinity spore outgrowth transcriptomics

***Correspondence:**
Dr. Ralf Moeller
Ralf.moeller@dlr.de

Keywords: *B. subtilis* spore germination, outgrowth, ripening, high salinity, osmotic stress, NaCl, RNA-seq

1 Supplementary figures

Figure S1

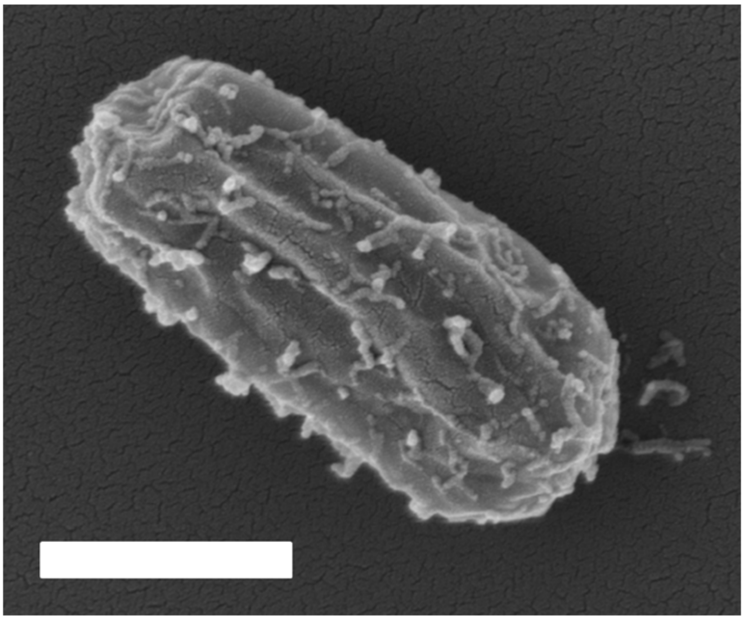

Fig. S1: SEM picture of a dormant *B. subtilis* spore. Scale bar = 500 nm.

Figure S2

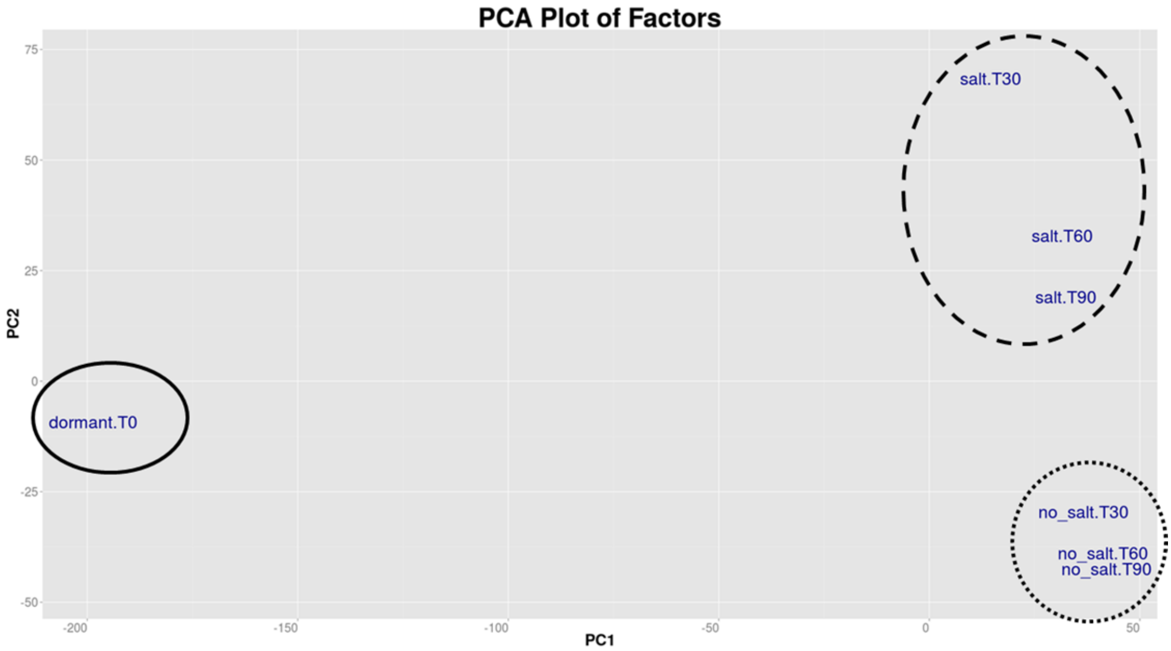

Fig. S2: Principal component analysis plot comparing the transcriptome of the tested sample time points (30, 60, and 90 min) consisting of two biological replicates each. Time points of the different conditions are circled: dormant spores (solid line), outgrowth in the presence of NaCl (dashed line), and outgrowth in the absence of NaCl (dotted line).

Figure S3

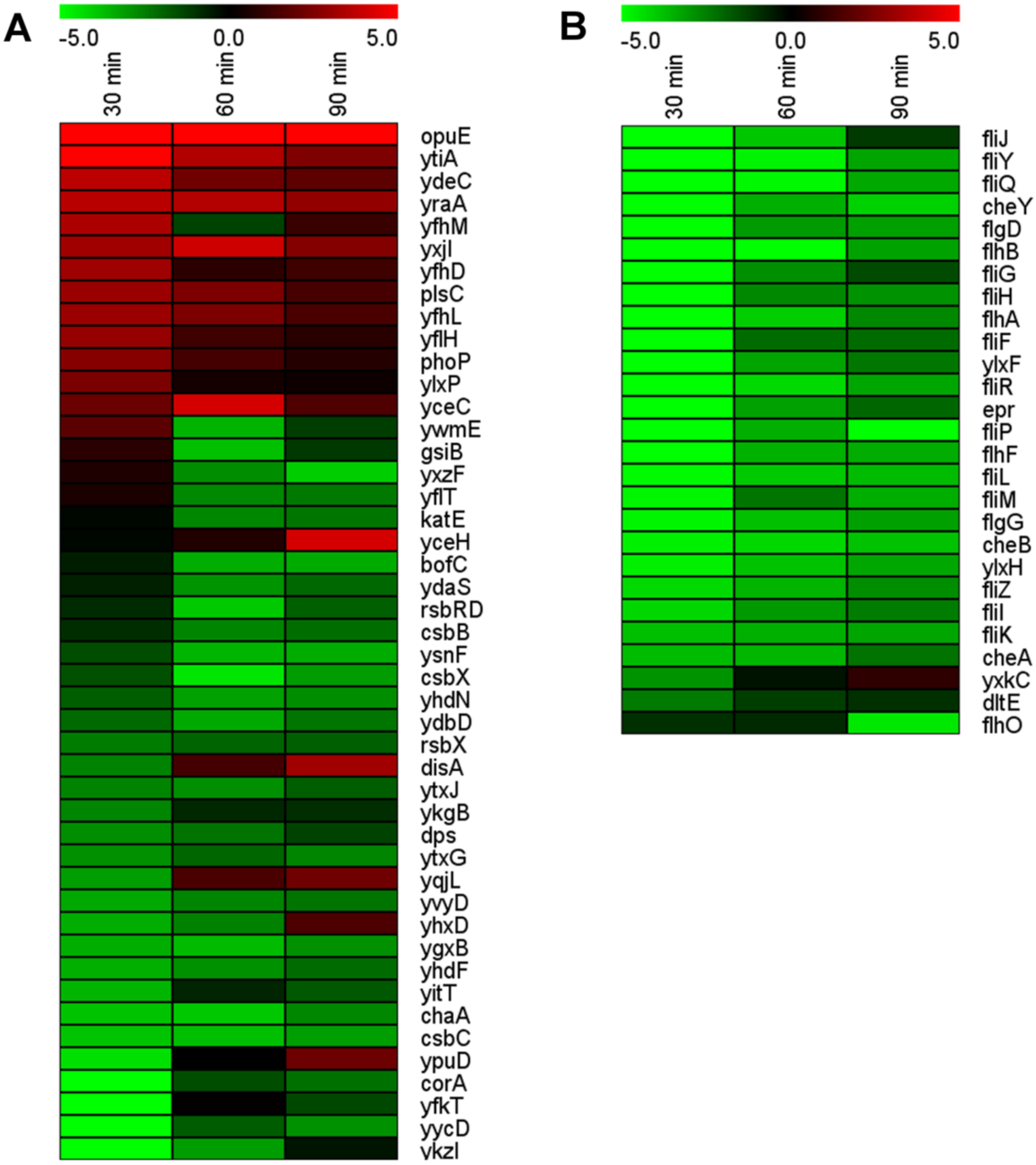

Fig. S3: Expression profiles of differentially expressed genes in (A) the σ^B regulon that governs the general stress response and (B) the σ^D regulon involved in motility and chemotaxis. Only significantly differentially expressed genes are shown. Cutoff values (log_2FC) of the color scale are indicated at the top of the figure.

2 Supplementary tables

Table S1: Functional categorization of differentially expressed genes (TopHits)

Category [a]	30 min		60 min		90 min	
	Up	Down	Up	Down	Up	Down
1.1 Cell wall & cell division	12	27	5	4	7	6
1.2 Transporters	42	61	29	34	22	23
1.3 Homeostasis	11	19	5	7	6	3
2.1 Electron transport & ATP synthesis	4	9	2	1	1	2
2.2 Carbon metabolism	19	22	5	10	3	10
2.3 Amino acid/nitrogen metabolism	15	103	17	29	12	52
2.4 Lipid metabolism	10	6	3	2	3	3
2.5 Nucleotide metabolism	13	21	12	9	12	5
2.6 Additional metabolic pathways	19	57	13	25	10	20
3.1 Genetics	10	21	3	2	1	0
3.2 RNA synthesis & degradation	2	7	2	2	1	1
3.3 Protein synthesis, modification & degradation	37	36	9	12	6	13
3.4 Regulation of gene expression	45	37	18	12	8	9
4.1 Exponential & early post exponential lifestyles	5	41	3	13	2	11
4.2 Sporulation & germination	19	34	5	9	11	6
4.3 Coping with stress	73	63	51	27	39	14
4.4 Lifestyles/miscellaneous	3	1	2	0	1	0
5.1 Prophages	13	19	4	7	4	5
5.2 Mobile genetic elements	0	1	0	0	0	0
6.1 Essential genes	31	27	1	7	4	11
6.2 Membrane proteins	88	133	49	62	37	46
6.3 GTP-binding proteins	0	1	0	0	0	1
6.4 Phosphoproteins	20	49	7	22	3	16
6.5 Universally conserved proteins	4	1	0	1	1	1
6.6 Poorly characterized/putative enzymes	11	26	5	8	5	8
6.7 Proteins of unknown function	66	90	32	27	19	17
6.8 Short peptides	0	2	0	0	0	0
6.9 ncRNA	1	0	1	0	1	0
6.10 Pseudogenes	1	3	0	0	0	0

[a] Transcriptomic data was categorized according to the *Subti*Wiki database (http://www.subtiwiki.uni-goettingen.de).

Table S2: Functional categorization of differentially expressed genes (HighFold)

Category [a]	30 min		60 min		90 min	
	**Up**	**Down**	**Up**	**Down**	**Up**	**Down**
1.1 Cell wall & cell division	0	5	1	1	0	2
1.2 Transporters	16	16	9	3	7	6
1.3 Homeostasis	2	1	3	2	2	1
2.1 Electron transport & ATP synthesis	1	0	0	0	0	0
2.2 Carbon metabolism	4	4	2	0	2	3
2.3 Amino acid/nitrogen metabolism	5	45	4	4	1	20
2.4 Lipid metabolism	0	0	0	0	0	0
2.5 Nucleotide metabolism	10	5	10	2	9	0
2.6 Additional metabolic pathways	2	7	4	4	2	2
3.1 Genetics	0	7	0	0	0	0
3.2 RNA synthesis & degradation	0	1	1	0	0	0
3.3 Protein synthesis, modification & degradation	3	6	1	4	1	0
3.4 Regulation of gene expression	8	7	4	1	0	1
4.1 Exponential & early post exponential lifestyles	1	17	0	1	0	1
4.2 Sporulation & germination	2	11	0	2	4	1
4.3 Coping with stress	22	11	13	1	7	2
4.4 Lifestyles/miscellaneous	1	0	1	0	1	0
5.1 Prophages	1	7	1	1	0	2
5.2 Mobile genetic elements	0	0	0	0	0	0
6.1 Essential genes	1	0	0	3	0	0
6.2 Membrane proteins	24	37	13	4	9	13
6.3 GTP-binding proteins	0	1	0	0	0	0
6.4 Phosphoproteins	5	12	2	1	1	3
6.5 Universally conserved proteins	0	0	0	0	0	0
6.6 Poorly characterized/putative enzymes	1	6	0	2	0	2
6.7 Proteins of unknown function	12	32	5	3	4	5
6.8 Short peptides	0	0	0	0	0	0
6.9 ncRNA	0	0	1	0	1	0
6.10 Pseudogenes	0	1	0	0	0	0

[a] Transcriptomic data was categorized according to the *Subti*Wiki database (http://www.subtiwiki.uni-goettingen.de).

Table S3: Selected *B. subtilis* regulons and their behavior during outgrowth at high salinity

Regulator	Regulator action	# of genes	Differentially expressed[a]		
			%	# up	#down
AhrC	Activates *roc* genes & represses rest of the regulon (arginine synthesis genes)	16	94	0	15
AzlB	Represses the regulon (branched-chain amino acid transport)	5	100	5	0
BirA	Represses the regulon (biotin synthesis)	7	71	0	5
BkdR	Activates the regulon	7	71	0	5
CcpN	Represses the regulon	4	100	4	0
CodY	Mainly (96 %) repression of the regulon	142	54	1	75
DegU	Mainly (85 %) activation of the regulon	26	19	1	4
GbsR	Represses the regulon	6	83	5	0
FatR	Represses the regulon	2	100	x	x
LutR	Represses the regulon (lactate utilization)	3	100	0	3
Fur	Represses the regulon (iron homeostasis)	50	34	0	17
GltC	Activates the regulon (glutamate synthase)	2	100	0	2
Gly-box	Termination (glycine utilization)	3	100	3	0
GntR	Represses the regulon (gluconate utilization)	4	100	4	0
HxlR	Activates the regulon	2	100	0	2
OhrR	Represses *ohrA* (organic hydroperoxide resistance protein)	1	100	1	0
PadR	Represses the regulon	3	100	3	0
preQ1 riboswitch	Termination (tRNA modification)	4	75	0	3
PucR	Mainly (94%) activation of the regulon	18	78	0	14
PutR	Activates the regulon (proline utilization)	3	100	0	3
PyrR	Antitermination (pyrimidine biosynthesis)	10	100	10	0
RocR	Activates the regulon (amino acid utilization)	7	100	0	7
S-Box	Termination	28	82	4	19
Stringent response	Mainly (96 %) repression of the regulon	121	31	24	13
T-Box	Antitermination	37	43	1	15
YxdJ	Activates the regulon	3	100	3	0
Zur	Represses the regulon	8	75	6	0
AdeR	Activation of *ald* (alanine dehydrogenase)	1	100	0	1
CitR	Represses *citA* (citrate synthase)	1	100	0	1
DesR	Activates *des* (fatty acid desaturase)	1	100	1	0
Thi-box	Termination	14	64	0	9
YdaO riboswitch	Termination in the presence of c-di-AMP	3	100	0	3
YfmP	Represses the regulon	2	100	2	0

[a] The numbers of up and downregulated genes only include genes that have the same expression direction (up/downregulated) at all three time points; x = differential behavior (up- and downregulation) at different sample time points

Table S4: Overlap of differentially expressed genes during outgrowth at high salinity and genes involved in other stress responses

Category [a]	# of genes[b]	Differentially expressed[c]		
		%	# up	#down
Cell envelope stress proteins	133	39	29	16
Acid stress proteins	5	20	1	0
Heat shock proteins	27	30	6	1
Cold stress proteins	16	19	3	0
Coping with hypo-osmotic stress	4	0	0	0
Resistance against oxidative & electrophile stress	63	22	4	9
Resistance against other toxic compounds (nitric oxide, phenolic acids, flavonoids, oxalate)	16	25	4	0
Resistance against toxic metals	22	9	1	1
Resistance against toxins/antibiotics	104	27	17	11
Biosynthesis of antibacterial compounds	55	27	4	11
Toxins, antitoxins & immunity against toxins	41	10	3	1

[a] Transcriptomic data was categorized according to the *Subti*Wiki database (http://www.subtiwiki.uni-goettingen.de).

[b] Number of genes within the regulon

[c] The numbers of up- (#up) and downregulated (#down) genes only include genes that have the same expression direction (up/downregulated) at all three time points. The percentage of differentially expressed genes includes all genes.

3 Supplementary datasets

Dataset S1: Overview of differential gene expression. The dataset includes significant differential gene expression information for each sample time point, an overview of all significantly differentially expressed genes (all time points), an overview of all genes including the ones that were not significantly changed, and the list of functional categories obtained from the *Subti*Wiki database (http://www.subtiwiki.uni-goettingen.de). [Separate .xls file (Nagler et al supplementary material 02.xlsx)]
